# Exo-erythrocytic development of *Plasmodium matutinum* (lineage pLINN1) in a naturally infected roadkill fieldfare *Turdus pilaris*

**DOI:** 10.1186/s12936-022-04166-x

**Published:** 2022-05-15

**Authors:** Helene Pendl, Carolina Hernández-Lara, Jakub Kubacki, Nicole Borel, Sarah Albini, Gediminas Valkiūnas

**Affiliations:** 1 Pendl Lab, Untere Roostmatt 7, 6300 Zug, Switzerland; 2 Nature Research Centre, Akademijos 2, 08412 Vilnius, Lithuania; 3 Institute of Virology, Vetsuisse Faculty, University of Zürich, Winterthurerstrasse 266a, 8057 Zurich, Switzerland; 4 Institute of Veterinary Pathology, Vetsuisse Faculty, University of Zürich, Winterthurer Strasse 268, 8057 Zurich, Switzerland; 5 Section of Poultry and Rabbit Diseases, Institute for Food Safety and Hygiene, Vetsuisse Faculty, University of Zürich, Winterthurerstrasse 270, 8057 Zurich, Switzerland

**Keywords:** *Plasmodium matutinum*, pLINN1, Birds, Exo-erythrocytic development, Virulence, Roadkill

## Abstract

**Background:**

Species of *Plasmodium* (Haemosporida, Plasmodiidae) are remarkably diverse haemoparasites. Information on genetic diversity of avian malaria pathogens has been accumulating rapidly, however exo-erythrocytic development of these organisms remains insufficiently addressed. This is unfortunate because, contrary to *Plasmodium* species parasitizing mammals, the avian malaria parasites undergo several cycles of exo-erythrocytic development, often resulting in damage of various organs. Insufficient knowledge on the exo-erythrocytic development in most described *Plasmodium* species precludes the understanding of mechanisms of virulence during avian malaria. This study extends information on the exo-erythrocytic development of bird malaria parasites.

**Methods:**

A roadkill fieldfare (*Turdus pilaris*) was sampled in Switzerland and examined using pathologic, cytologic, histologic, molecular and microbiologic methods. Avian malaria was diagnosed, and erythrocytic and exo-erythrocytic stages of the parasite were identified using morphologic characteristics and barcode DNA sequences of the cytochrome *b* gene. The species-specific characteristics were described, illustrated, and pathologic changes were reported.

**Results:**

An infection with *Plasmodium matutinum* lineage pLINN1 was detected. Parasitaemia was relatively low (0.3%), with all erythrocytic stages (trophozoites, meronts and gametocytes) present in blood films. Most growing erythrocytic meronts were markedly vacuolated, which is a species-specific feature of this parasite’s development. Phanerozoites at different stages of maturation were seen in leukocytes, macrophages, and capillary endothelial cells in most organs examined; they were particularly numerous in the brain. Like the erythrocytic meronts, growing phanerozoites were markedly vacuolated. Conspicuous exo-erythrocytic development and maturation in leucocytes suggests that this fieldfare was not adapted to the infection and the parasite was capable to escape from cellular immunity.

**Conclusions:**

This is the first report of exo-erythrocytic development of the malaria parasite lineage pLINN1 during single infection and the first report of this lineage in the fieldfare. The findings of multiple phanerozoites in brain, skeletal muscle, and eye tissue in combination with signs of vascular blockage and thrombus formation strongly suggest an impaired vision and neuromuscular responsiveness as cause of the unexpected collision with a slowly moving car. Further studies on exo-erythrocytic stages of haemosporidian parasites are pivotal to understand the true level of populational damage of avian malaria in wild birds.

## Background

Avian malaria pathogens of the genus *Plasmodium* (Plasmodiidae, Haemosporida) are cosmopolitan, with over 50 species described [1] and many more different genetic lineages determined (MalAvi database, http://130.235.244.92/Malavi, accessed February 2022). As these parasites are present in the peripheral blood circulation, they are easy to access for morphologic and genetic studies, which during the past 25 years resulted in a prominent increase of knowledge on various aspects of their genetic diversity as well as geographic and host distribution [2]. Development of sensitive and easy to use genetic markers considerably improved opportunities for pathogen diagnostics and ecologic research. However, information on an important part of the life cycle of malaria parasites and related haemosporidians (Haemosporida)–the exo-erythrocytic development–still remains at an early stage. This is particularly true for wild birds due to the difficulties in accessing these stages for research, which requires animal dissection, direct investigation of organs and application of histologic techniques [3, 4].

To date, the exo-erythrocytic development remains non-described or fragmentarily known in most avian haemosporidian parasite species. This is unfortunate because, contrary to malaria parasites of humans and other mammals, avian *Plasmodium* species undergo several cycles of exo-erythrocytic merogony and can markedly damage organs before they acquire the ability to infect red blood cells. Furthermore, secondary exo-erythrocytic development can be initiated by merozoites from erythrocytic meronts [5–7]. This complicates understanding of the development of avian malaria parasites in avian hosts and makes disease prognoses to be speculative even during low chronic malaria parasitaemia [8, 9]. Numerous experimental observations show that the secondary exo-erythrocytic meronts (phanerozoites) might lead to severe disease and even mortality of birds [5, 6, 10, 11]. However, most experimental studies deal with non-adapted host-parasite associations, which could bias the understanding of the true virulence of the same pathogen in natural populations. Information on the exo-erythrocytic development of haemosporidian parasites in naturally infected birds would be a valuable supplement to experimental observations, but currently remains insufficient in wildlife [4, 12, 13]. This study provides first information on a natural infection with *Plasmodium matutinum* (genetic lineage pLINN1) in a wild fieldfare (*Turdus pilaris*). This parasite lineage is common in European species of thrushes (Turdidae) and flycatchers (Muscicapidae), and it seems to be transmitted across the Holarctic [13, 14].

## Methods

### Case history

On September 7th, 2021, one of the authors (HP) witnessed a roadkill of a fieldfare (*Turdus pilaris*) close to a nature reserve in the Canton Zug, Switzerland (47° 13’ 38.4" N, 8° 24’ 21.4" E). The bird flew frontally into the grille of a slow-moving car and fell into the road ditch in front of the author. First examination confirmed the death of the bird with multiple fractures visible at the frontal part of the head and the thorax. Permission of collection of the carcass was given by telephone by the Office of Forestry and Game of the Canton Zug.

### Necropsy, cytology, histology

Necropsy was performed following a standardized protocol [15] approximately two hours after collection. A full organ set was preserved comprising samples from the cardiovascular (heart, heart blood), the respiratory (lung, air sacs), gastrointestinal-hepatic (oropharynx, crop, proventriculus, ventriculus, intestine, pancreas, cloaca, liver), urogenital (kidney, ovary, oviduct), endocrine (thyroid gland), hematopoietic (spleen, bone marrow, thymus), musculo skeletal (pectoral and femoral muscles, femoral bone, skull bone), and central nervous and sensory system (all parts of the brain including the eyeballs). Samples were split into halves with one part frozen at − 20° for virology/molecular diagnostics and the other part fixed in buffered formalin for histology (Formafix™ Switzerland AG, Stationsstr. 3, CH 8335 Hittnau). Small samples were preserved in formalin only.

Cytologic samples were taken from fresh material with swab-roll imprint preparation from conjunctiva, cloaca, air sacs, and intestinal contents, with blood film technique from fluidy parts of heart and lung, with imprints or scrape-squash preparations from liver, lung, spleen, kidney, myocardium, feather quills, brain, thyroid gland, pectoral and femoral muscle. The imprint preparations of the brain were taken from the material protruding from the skull fractures close to the third eyelids. Drops of heart and lung blood were dried and stored on filter paper for molecular diagnostics. Cytologic samples were stained with a one-step Wright-Giemsa-Protocol and mounted with Entellan New™ [16]. Formalinized samples were embedded in paraffin, and histologic sections of 2 μm thickness were prepared in Hematoxylin-Eosin (HE), Periodic-Acid-Shift (PAS), and Prussian Blue (PB) stain according to standard techniques at the Fachpraxis für Tierpathologie, Hartelstraße 30, D-80689 Munich, Germany (https://www.tierpathologie-muenchen.de).

Histopathologic and cytopathologic evaluation of the samples was carried out with an Olympus BX41 light microscope under × 40, × 100 and × 1000 magnification. Photographic documentation was performed with a ProgRes^®^ C10 Plus digital camera and ProgRes^®^ CaptivePro v2.8.8 imaging software from Jenoptik Optical Systems GmbH, Germany.

### Parasitology

#### Microscopic examination of blood films

Examination of stained films from heart and lung blood was performed using an Olympus BX51 light microscope equipped with an Olympus DP12 digital camera and Olympus DP-SOFT imaging software. Per blood film, 100 microscope fields were scanned at high magnification (× 1000) to prepare images of parasites and estimate parasitaemia intensity of erythrocytic stage. The latter was determined by counting the actual number of parasites in 2000 erythrocytes and expressed as percentage according to [17]. Phanerozoite parasitaemia intensity was estimated using the similar methodology. Mainly, the actual number of single observed phanerozoites, which were seen after screening of a portion of blood smear containing 2000 erythrocytes, were counted. Morphologic identification of the parasite species was carried out on blood films at high magnification according to [6]. Exoerythrocytic stages were measured using ImageJ 1.53a software (National Institutes of Health, Bethesda, MD, USA, https://imagej.nih.gov/ij/USA; accessed on 21 October 2021) [18]. Voucher parasite preparations containing blood stages (accession number of blood slides 49394NS and 49395NS) and tissue meronts (accession numbers of cytologic and histologic preparations 49396–49403 NS) were deposited at Nature Research Centre, Vilnius.

#### DNA extraction, PCR and sequencing for malaria parasite molecular characterization

DNA was extracted from the drops of heart and lung blood and re-thawed tissue samples from the frozen retained samples stored on filter paper using an ammonium acetate protocol [19]. Then, a standard nested PCR protocol was applied to identify the cytochrome *b* lineage [20, 21]. Primers HaemNFI/HaemNR3 and HaemF/HaemR2, as well as parameters of PCR, were the same as those described in the original protocol. Positive (*Plasmodium* sp.) and negative (nuclease-free ddH_2_O) controls were included. PCR products were run on a 2% agarose gel to check for positive amplifications, which were sequenced from 3’ and 5’ ends with Big Dye Terminator V3.1 Cycle Sequencing Kit and ABI PRISMTM 3100 capillary sequencing robot (Applied Biosystems, Foster City, CA, USA). Cytochrome *b* mitochondrial gene sequences (479 bp) quality and presence of mixed infections (double peaks) was assessed using SnapGene Viewer 5.2.4 software (Insightful Science, San Diego, CA, USA, www.snapgene.com; accessed on 20 November 2021). Lineage identification was carried out by BLAST searches in MalAvi [22] and GenBank databases with Megablast algorithm (www.ncbi.nlm.nih.gov/genbank/; accessed on 10 October 2021). Obtained DNA sequence information was compared with results of microscopic parasite identification.

### Microbiology

Microbiology diagnostics to detect possible concurrent infections were performed at the Vetsuisse Faculty, University of Zurich.

#### Chlamydiaceae

Extracted DNA from liver, heart, brain, lung, spleen, kidney and ventriculus were screened for *Chlamydiaceae* infections using the 23 S rRNA *Chlamydiaceae*-specific real-time PCR, resulting in an amplicon of 111 base pairs and using primers Ch23S-F, Ch23S-R and probe Ch23S-p [23]. Internal positive controls included enhanced green fluorescent protein (eGFP) [24].

#### West Nile Virus, Usutu Virus

Additional PCRs for West Nile Virus (WNV) and Usutu Virus were run on RNA extracts from frozen liver samples. WNV reverse transcriptase real-time PCR was carried out according to a modified protocol by Eiden et al. [25] (modification and validation thereof done at the Institute of Virology and Immunology, Mittelhäusern) detecting WNV lineage 1 and 2 genomes and including an internal amplification control (eGFP-PCR) [25, 26]. The 25 µl reaction mix contained 12.5 µl “2x QuantiTect Probe RT-PCR Master Mix” (Qiagen), 400nM of forward primer WNV_Eiden_mod_F (5‘AGAAGTTCGTCTGCGTGAGC3’), 400nM of reverse primer WNV_Eiden_mod_R (5‘GCCCTCCTGGTTTCYTAGA3’) and 200nM of probe WNV_Eiden_mod_P (5‘FAMTGACAAACTTAGTAGTGTTTGTGAGGATT-TAMRA3’), 0.25 µl “QuantiTect RT Mix” (Qiagen) and 5 µl sample RNA. Usutu Virus reverse transcriptase real-time PCR was essentially done as described in [27]. All primers and probe were purchased from Microsynth AG, Balgach, Switzerland. All PCR reactions were run on a “7500 Fast RealTime PCR System” (Thermo Fisher Scientific), with the standard cycle protocol: 30 min at 48 °C, 10 min at 95 °C, then 45 times: 15 s at 95 °C, 1 min at 53 °C, 1 min at 70 °C. All samples were analysed in duplicates.

### Bacteriological culture

Thawed frozen liver tissue was cultured using tryptone soy broth, Columbia agar with 7% sheep blood and bromothymolblue-lactose agar (Oxoid / Thermo Fisher Scientific, Waltham, MA, USA), incubated aerobically for 48 h at 37 °C.

#### Next generation sequencing for whole virome

Frozen organ samples were pooled in four batches for Next Generation Sequencing (NGS) with batch 1 containing lungs and heart, batch 2 containing liver, batch 3 containing ventriculus, and batch 4 containing intestines. The organ samples were prepared according to previously established ViroScreen protocol at the Virology Institute of the University of Zurich, Switzerland [28]. Briefly, 30 mg of homogenized by scalpel organs were diluted in 270 µl PBS and homogenized in the TissueLyser (Qiagen) for 2 min at 20 Hz. Then, the samples were enriched for viral nucleic acid, amplified by sequence-independent single primer amplification and sequencing libraries have been constructed. Sequencing was performed at Functional Genomics Center Zurich (ETH, Zurich, Switzerland) on the Illumina NovaSeq machine in a 2 × 150 bp read length run. The raw sequencing reads (from 2.7 to 18.7 million reads per sample) were quality controlled and aligned in reference guided analysis and *de novo* assembly pipelines as described previously [29]. In summary, quality-controlled reads were aligned to an inhouse database containing 61,620 complete viral genomes downloaded from the NCBI database, and assembled using megahit (version 1.1.3) with multiple k-mers [30] and metaspades (v3.12.0) [31].

## Results

### Necropsy

The bird was a female fieldfare with adult plumage and in slight hypertrophy of pectoral muscle condition (grade 2–3 at a semiquantitative scale from 0 = cachectic to 4 = obese) [32]. The inner secondary feathers were not grown to full-length most likely due to seasonal moulting. Search for ectoparasites was negative. The bird weighed 79 g, which is slightly underweight according to data available online (Birds of Switzerland, https://www.vogelwarte.ch/en/birds/birds-of-switzerland/fieldfare, accessed 10 January 2022). *Rigor mortis* was absent. External examination revealed multiple open fractures of the sternum and the frontal skull between the eyes with prominent haemorrhages and brain tissue protruding externally from under the third eyelids. Feathers around the cloaca were slightly soiled with yellowish, opaque fluid identical with the liquid content of the cloaca. Internal examination revealed a longitudinal rupture of the heart and multiple ruptures in the lungs with prominent amounts of blood dispersed into the surrounding cavities. The intestines were filled with yellowish, opaque, seromucous fluid. Spleen, liver, and kidney were slightly enlarged and swollen. Serosal surfaces were thin, clear, and translucent. The brain showed signs of prominent haemorrhage and tissue disintegration in the frontal area of the cerebrum as well as haemorrhages in the occipito-basal part of the cerebellum suggesting an additional *contre coup* lesion.

### Parasitology

Cytologic and histologic examinations revealed intracellular stages of *P. matutinum* in many tissues, with lung and brain being the most affected organs with a disseminated to diffuse distribution pattern. Lesser amounts of parasitic stages in oligofocal distribution were detected in the myocardium, the *Pecten oculi*, and skeletal muscle tissue (periocular, pectoral, and femoral muscle). Low parasitic load, often only detectable as single findings in cytologic preparations, was seen in peripheral blood, spleen, bone marrow, gastrointestinal tract, liver, and kidney. Parasites were easily detected in cytologic preparations, whereas in histologic preparations a clear visualization of the parasites often was hampered by the relative thickness of the tissue section compared to the monolayer of the cytologic specimens. Multiple scanning of the same view in various planes of focus was necessary for visualization and particularly difficult in haemic cells.

#### Description of erythrocytic stages

Parasitaemia was 0.3%. Most erythrocytic stages of *P. matutinum* were seen in thin films prepared from heart and lung blood with rare additional findings in the bone marrow. PCR diagnostics detected the lineage pLINN1 of this parasite (GenBank accession OL653715) and confirmed the morphologic identification. A single haemosporidian infection was present.

**Fig. 1:**
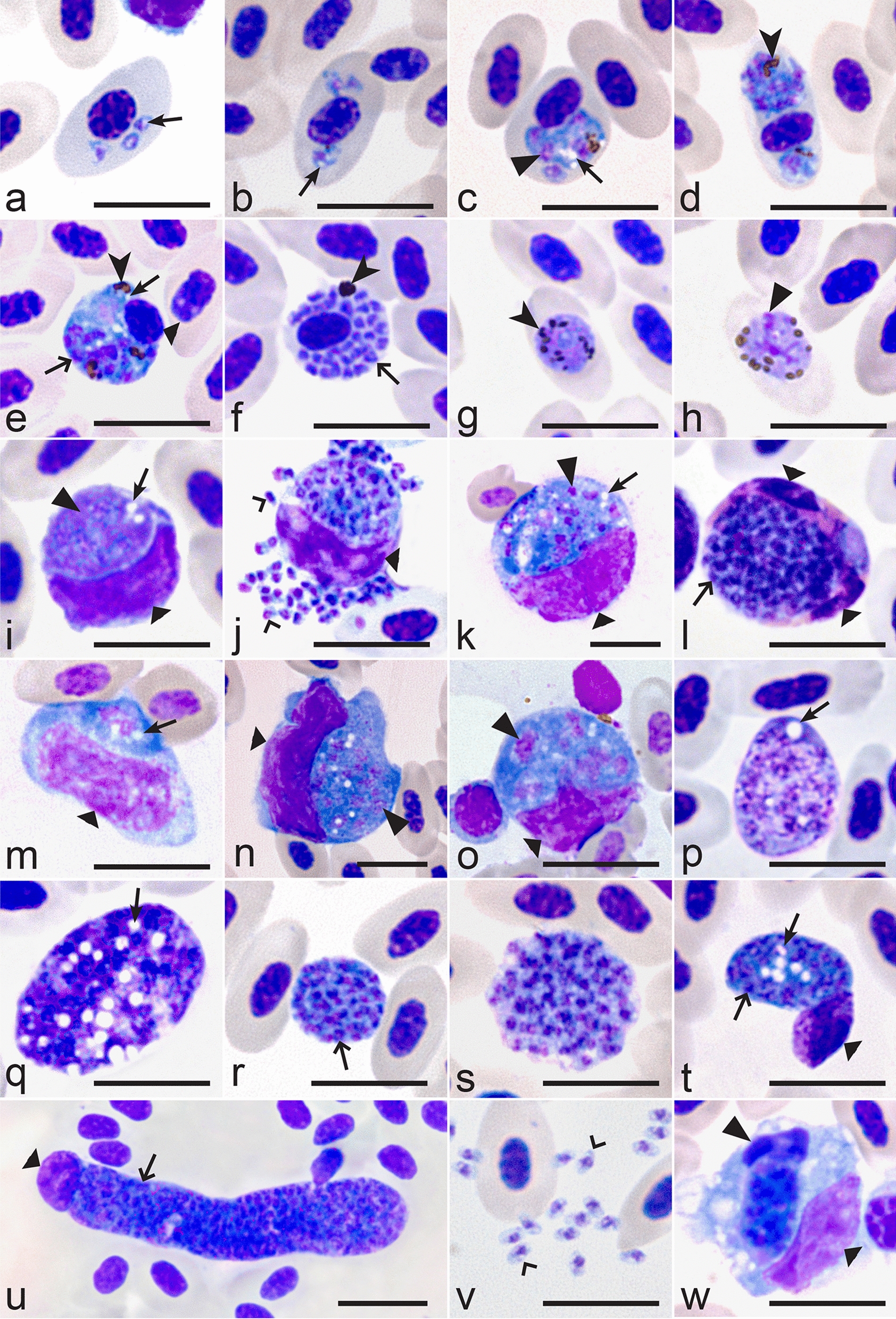
Blood stages of *Plasmodium* (*Haemamoeba*) *matutinum* (lineage pLINN1) from a roadkill fieldfare *Turdus pilaris*: erythrocytic trophozoites (**a, b**), erythrocytic meronts (**c–f**), macrogametocyte (**g**), microgametocyte (**h**), phanerozoites (**i–u, w**) and phanerozoic merozoites (**v**). Multiple infection of same host cell with several parasites was common (**a, b, d**). Erythrocytic meronts often contained prominent vacuoles (**c, e**). Mature gametocytes often enucleated infected erythrocytes (**g, h**). Developing (**i, k**) and mature (**j**) phanerozoites in mononuclear leucocytes; note the prominent cytoplasm, nuclei, and vacuoles (**i, k)** and numerous mature phanerozoic merozoites, each containing a prominent nucleus and cytoplasm (**j**). Mature phanerozoite in a granulocyte (**l**); note the numerous nearly mature merozoites. Developing phanerozoites in mononuclear leucocytes (**m-o**); note the prominent vacuoles, which numbers increase as the parasites increase in size (compare **m** with **n, o**). Extracellular phanerozoites in heart blood at different stages of maturation (**p-s**); note that vacuolization of the cytoplasm decreases in maturing phanerozoites (compare **p, q** with **r, s**). Extracellular developing (**t**) and nearly mature phanerozoite (**u**), which are normally located in endothelial cells of capillaries (see Fig. 2b) but were washed out from the capillaries and present in the heart blood as free bodies; note numerous developing merozoites and still adjacent host-cell nucleus. Mature phanerozoic merozoites (**v**); note oval shape of the parasites containing prominent nuclei and cytoplasm. Phanerozoite phagocytized by a mononuclear leucocyte (**w**) indicating an immune reaction against free phanerozoites *intra vitam*; note the degenerating nuclei and cytoplasm of the affected parasite. All images taken from cytologic preparations of heart blood in Wright Giemsa stain, except for the images **m** and **o** (cytologic imprint of the lung in Wright Giemsa stain). *Simple arrows* vacuoles, *triangle arrowheads* parasite nuclei, *simple arrowheads* pigment granules, *simple wide short arrows* developing merozoites, *triangle wide arrowheads* host cell nuclei, *simple wide long arrows* parasites, *simple wide arrowheads* mature merozoites. *Scale bars* 10 μm

Trophozoites (Fig. 1a, b), growing (Fig. 1c–e) and mature (Fig. 1f) erythrocytic meronts and gametocytes (Fig. 1g, h) were seen. These were present mainly in mature erythrocytes, but trophozoites were also seen in immature, polychromatic erythrocytes. Multiple infection of one erythrocyte with several growing parasites was common (Fig. 1a, b, d). Each early trophozoite possessed a prominent nucleus and a readily visible centrally located vacuole within the cytoplasm (Fig. 1a). In growing trophozoites, the amount of nuclear material and cytoplasm was slightly increased, and two small vacuoles and a pigment granule were visible (Fig. 1b). Growing meronts were characterized by prominent nuclei and cytoplasm with several readily visible vacuoles (Fig. 1c, e). With increasing maturation, the size of the nuclei, the amount of cytoplasm and the number of vacuoles decreased, but the number of pigment granules increased. Mature meronts contained up to 30 merozoites. Pigment granules were gathered in a solid mass, and vacuoles were absent (Fig. 1f). Mature gametocytes were roundish, contained prominent nuclei and roundish or slightly oval pigment granules. These stages were often present in enucleated erythrocytes (Fig. 1g, h). Other morphologic details of blood stages coincided with former parasite descriptions [6, 14] and are not repeated here.

#### Description of exo-erythrocytic stages

**Fig. 2:**
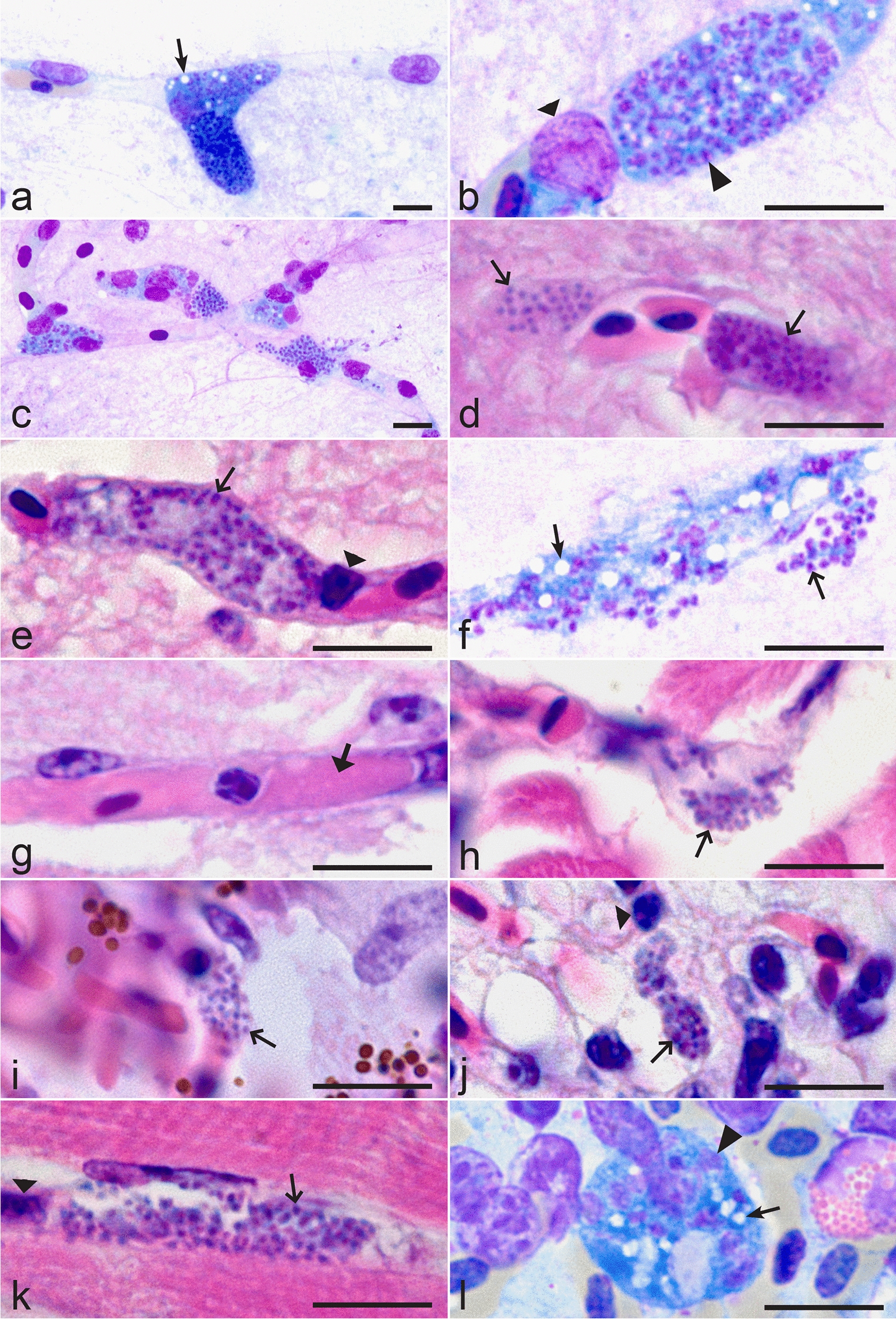
Phanerozoites of *Plasmodium* (*Haemamoeba*) *matutinum* in endothelial cells of brain (**a–f**), eye (**h, i**), lungs (**j**), pectoral muscle (**k**) and kidney (**l**). Two phanerozoites in different stages of maturation in a capillary of the frontal telencephalon **(a**); note that the younger phanerozoite (top) contains more vacuoles, larger nuclei and more cytoplasm than the nearly mature phanerozoite (bottom). Maturing phanerozoite in a capillary of the frontal telencephalon (**b**); note that the parasite completely blocks the capillary. Four phanerozoites at different stages of maturation in capillary endothelial cells of the frontal telencephalon (**c**); note the signs of cellular disintegration and nuclear fragmentation, whose exclusive occurrence in close neighbourhood to phanerozoites supports vascular blockage as cause of disintegration. Two phanerozoites in different stages of maturation in capillary endothelial cells of the molecular layer of the cerebellum (**d**); congestion of erythrocytes is visible. Maturing phanerozoite in a capillary endothelial cell of the mesencephalon (**e**). Mature phanerozoite in the frontal telencephalon (**f**); note that vacuolization is still visible in the maturing parasite. Fibrinoid microthrombosis in a capillary of the cerebellum molecular layer (**g**). Phanerozoite in a capillary endothelial cell of the endomysium of an oculomotoric skeletal muscle fibre, which is adjacent to the ocular bulb (**h**). Phanerozoite within a capillary endothelial cell of the *Pecten oculi* (**i**). Phanerozoite in capillaries of lung (**j**) and pectoral muscle (**k**); note the closely located nuclei of host cells. Phanerozoite in the kidney (**l**); note the markedly vacuolated cytoplasm. Images taken from cytologic imprints in Wright-Giemsa stain (**a–c, f, l**) and histologic preparations in hematoxylin & eosin stain (**d, e, g–k**). *Simple arrows* vacuoles, *triangle arrowhead* nuclei of developing phanerozoites, *triangle wide arrowheads* host cell nuclei, *simple wide arrows* developing merozoites, *triangle arrow* microthrombus *Scale bars* 10 μm

In addition to erythrocytic stages (see description above), numerous phanerozoites (Fig. 1i–u) at different stages of maturation and mature merozoites (Fig. 1j, v) were detected in blood films prepared from heart and lung blood. Phanerozoite parasitaemia intensity was approximately 0.1%. Apart from few granulocytes (Fig. 1l), the majority of phanerozoites were seen in mononuclear cells morphologically most likely pertaining to the mononuclear-phagocyte-system (MPS) of monocytes and macrophages (Fig. 1i–k, m–o). The largest phanerozoites reached 20 μm in diameter (Fig. 1k, n, o). Furthermore, extracellular roundish phanerozoites (Fig. 1p–s) and free mature phanerozoic merozoites (Fig. 1v) were common in the blood films, most likely resulting from either artificial destruction of host cells during blood film preparation or true rupture *intra vitam*. Their morphology was similar to equivalent stages located intracellularly. However, phanerozoites can be readily distinguished due to absence of pigment granules, which develop only in erythrocytic stages in malaria parasites. Additionally, phanerozoites were significantly bigger than erythrocytic meronts (compare Fig. 1d, e and p–s). Interestingly, large (up to 47 μm in length), elongate, nearly mature phanerozoites also were seen in the circulation (Fig. 1t, u). Some of them were phagocytized by mononuclear cells (Fig. 1w). Such stages normally occur in endothelial cells of capillaries (see Fig. 2a–e).

Like in the erythrocytic meronts, the young phanerozoites developing in immune cells were markedly vacuolated and possessed prominent nuclei and abundant amounts of cytoplasm (Fig. 1i, k, m–o). The conspicuous, mainly circular vacuoles were variable in size with the largest vacuoles reaching 1.5 μm in diameter. The amount of the cytoplasm and size of nuclei decreased as parasites matured, and vacuoles were absent in mature phanerozoites (Fig. 1j, l). Mature merozoites were roundish or oval bodies with a readily visible portion of cytoplasm and centrally located nuclei (Fig. 1v). They were 1.6 ± 0.2 μm in biggest diameter.

Elongate phanerozoites (Fig. 2a–f, h–k) were exclusively seen in capillary endothelial cells of affected organs. They were particularly numerous in brain (Fig. 2a–f) and lung tissue with up to five phanerozoites per × 400 view field. Moderate (histology) to focally high (cytology) numbers were visible in the myocardium and pectoral muscle (Fig. 2k). The parasite followed the shape of capillaries, which often were completely blocked by large parasites (Fig. 2a–e, i–k). Both roundish and elongate phanerozoites were seen in lungs and kidneys (Fig. 2j, l). Elongate young phanerozoites were markedly vacuolated with prominent nuclei and conspicuous amounts of cytoplasm. Vacuolization, nuclear size, and cytoplasmic amount decreased as the parasites matured. This is particularly well visible in Fig. 2a, which shows two adjacent elongate phanerozoites at different stages of maturation. Vacuoles were seen in some nearly mature phanerozoites (Fig. 2f). Blockage of vascular lumina with congestion (Fig. 2d) up to thrombus formation with cellular disintegration, nuclear fragmentation and fibrin deposition (Fig. 2c, g) was observed in brain, lung, myocardium, and skeletal muscle.

For the first time in an avian malaria case, phanerozoites were detected in endothelial cells of eye tissues. Mature stages were present in the endomysium of an oculomotoric skeletal muscle fibre (Fig. 2h) and within the *Pecten oculi* (Fig. 2i).

### Cytology, Histology, Microbiology

A differential leukocyte count performed on 200 blood cells in the heart blood film revealed 69% mononuclear cells, 25% heterophilic granulocytes, and 6% eosinophilic granulocytes. The mononuclear cells consisted of both small and large lymphocytes as well as monocytes, all of which showed cytomorphologic features of reactivity such as increased cytoplasmic basophilia, vacuolation, bleb formation and a rather smooth delicate nuclear chromatin pattern. This similarity of morphology particularly hampered the differentiation of equally sized large lymphocytes and monocytes, which therefore were categorized in one mononuclear cell portion. The erythroid line was characterized by a moderate left shift with regular appearance of intravascular mitoses and disproportionately high numbers of very immature stages with round cell shape, high nucleo-cytoplasmic (N/C) ratio and deep basophilic cytoplasm. A corresponding left shift of the erythroid line in the bone marrow was present. In addition to the above-mentioned phagocytosis of parasitic stages, erythrophagocytosis by monocytes was regularly seen. A severe mononuclear infiltration was seen in the cytologic preparations of lung tissue with particularly high numbers of larger cells with cytologic characteristics of plasma cells, large lymphocytes and cells of the monocyte/macrophage system.

Mild to moderate lymphohistiocytic to lymphoplasmacytic inflammation with signs of lymphoid necrosis was seen in histologic sections of lung, liver, skeletal muscle, smooth muscle of the gizzard, as well as myo-, epi- and pericardium. The poorly demarcated infiltrates were present as perivascular cuffs or showed multifocal to disseminated interstitial distribution. In addition, the lung was moderately congested with mild fluid accumulation in aerated spaces and multifocal, mild to moderate perivascular oedema in beginning organization. A single, small, well-demarcated granuloma surrounding small nematode cross-sections was present.

In the spleen, prominent proliferation and nuclear degeneration (possibly due to beginning autolysis) was seen in the ellipsoidal reticular cells of the Schweiger-Seidel sheaths (SSS). The surrounding periarteriolar white pulp (PWP) contained a prominent amount of plasma cells, many of which also showed mild signs of autolysis/necrosis. There was a diffuse accumulation of golden-brown to blackish-brown pigment, which was mostly located intracellularly and partially birefringent under polarization. Search for parasitic structures revealed only very few intra-erythrocytic stages in cytology and was negative in histology.

Mild nematodiasis (*Capillaria* spp.*)* and cestodiasis with a single longitudinal section of each helminth was found in the intestinal lumen with mild signs of lymphoplasmacytic reactivity in the villous cores of the adjacent intestinal mucosa.

Molecular testing for *Chlamydiacaeae*, West Nile and Usutu Virus, and bacterial culture from liver tissue was negative. In the NGS, neither in the reference alignment to a database containing 61,620 complete viral genomes nor in *de novo* assembly viral reads/contigs were detected.

## Discussion

The key results of this study comprise (i) the first report and description of exo-erythrocytic development of the *P. matutinum* lineage pLINN1 during single infection, (ii) the first report of this infection in the fieldfare, and (iii) the first report of phanerozoite development in ocular structures, suggesting that this infection contributes to avian road kills due to impaired vision.

*Plasmodium matutinum* (pLINN1) is common in palearctic birds with a broad geographic distribution. Molecular characterization was developed using blood stages of the parasite isolated from the Thrush nightingale (*Luscinia luscinia*) [14]. This lineage is particularly common in species of the genus *Turdus* in Europe with the Common blackbird (*Turdus merula*) being the most common host [13]. This study expands the range of natural avian hosts for this malaria infection by the fieldfare, a migratory bird species with broad palearctic distribution [33].

Blood stages of *P. matutinum* can be readily distinguished due to marked vacuolization of the cytoplasm in trophozoites and growing meronts (Fig. 1a–e). Additionally, erythrocytic merogony is markedly synchronized, with meront maturation peaking in the morning and the cycle of the merogony being close to 24 h [5, 6, 34]. Vectors of the lineage pLINN1 are currently unknown, but likely to be *Culex* mosquitoes, as they were shown to be competent vectors of unidentified lineages of *P. matutinum* in America and Europe [11, 35, 36]. This parasite lineage was found in naturally infected *Culex* mosquitoes in Italy [37], however it remains to be proved that this parasite lineage completes sporogony and develops sporozoites in these mosquitoes.

To date, a primary exo-erythrocytic development has not been described for any strain of *P. matutinum* [5, 6, 14]. This paper describes for the first time a secondary exo-erythrocytic cycle with characteristic development of phanerozoites after a natural infection in a wild bird for the lineage pLINN1 during single natural infection. The observed morphology and location of the exo-erythrocytic meronts were indistinguishable from those seen in canaries (*Serinus canaria*) experimentally infected with unknown lineages of *P. matutinum* isolated from the Common blackbird and redwing in Italy [5, 6, 10] and the Common blackbird in Switzerland [11]. Previous studies reported the presence of phanerozoites in reticulo-endothelial cells of brain, liver, spleen, kidneys, lungs, heart muscle and bone marrow. According to our case report this list must be complemented with ocular structures and skeletal muscle. Furthermore, massive infection of circulating leucocytes and tissue macrophages was seen for the first time during this infection.

Characteristic features of the phanerozoite development of *P. matutinum* include the development of roundish phanerozoites in large mononuclear cells and occasionally granulocytes, the presence of elongate phanerozoites in endothelial cells of capillaries in various organs with particular prominence in brain and lung; the presence of conspicuous vacuolization in the cytoplasm of immature phanerozoites, the development of usually > 100 merozoites in mature phanerozoites with up to > 300 merozoites in the largest phanerozoites. The latter feature together with the presence of phanerozoites in brain tissue distinguishes *P. matutinum* from the morphologically similar malaria parasite *Plasmodium giovannolai*, which also parasitizes species of the genus *Turdus* in Europe [5, 6, 38, 39] but remains non-characterized molecularly [13]. It is worth to mention that numerous elongate phanerozoites were seen in blood films (Fig. 1u). This suggests that mature large phanerozoites might be washed out from fixed tissues into the circulation during intense *P. matutinum* infection, but this observation remains speculative due to possible mechanical impact. However, G. Valkiūnas (unpublished, pers. obs.) has observed occasionally similar structures in blood films of Common blackbirds, whose were naturally infected with *Plasmodium* sp. in Europe, indicating that such process might occur naturally and may be worth more attention of researchers. The biological meaning of this phenomenon remains unclear.

Conspicuous circular vacuoles were described in phanerozoites and/or erythrocytic meronts of *P. matutinum, P. giovannolai, Plasmodium griffithsi, Plasmodium lutzi* and *Plasmodium tejerai*, which belong to the subgenus *Haemamoeba* [5, 6]. Similar vacuoles have been reported in zygotes, ookinetes, early oocysts and gametocytes of many species of haemosporidian parasites belonging to the families Plasmodiidae, Haemoproteidae, Leucocytozoidae and Garniidae [6, 14, 40, 41]. The origin and function of such vacuoles, however, remains insufficiently understood. It is believed that they contain material, which plays a role in energy metabolism and is involved in the lipid metabolism of actively growing parasites. Similar to other fatty structures, this material might be washed out during alcohol fixation leaving a vacuole-like space in stained samples [6, 7, 14, 42]. Further studies are needed to elucidate the true nature of these structures. Absence of vacuoles in mature phanerozoites in *P. matutinum* suggests that vacuolization is a feature of immature, growing phanerozoites.

Himmel et al. [43] investigated the occurrence of various haemosporidian infections in a large sample of Eurasian blackbirds and song thrushes (*Turdus philomelos*) whose were found dead in Austria. Co-infections of various haemosporidians predominated in these samples, and numerous new lineages of *Plasmodium* parasites were found. It was shown that *P. matutinum* (pLINN1) often caused high exo-erythrocytic meront intensities in various organs. With the presence of cytomeres in maturing exo-erythrocytic meronts and the absence of conspicuous vacuolization in most of the illustrated exo-erythrocytic meronts in *P. matutinum* (pLINN1) two unusual characters of the exo-erythrocytic stages were reported. These features were not observed (cytomeres) or not characteristic (absence of vacuoles in growing meronts) in our study, which was based on a single *P. matutinum* (pLINN1) infection. These observations were in accordance with former studies dealing with this parasite morphospecies [5, 6, 10, 11, 35]. It is difficult to rule out that some of the described exo-erythrocytic stages, which were attributed to pLINN1 [43] might belong to other *Plasmodium* lineages, which could occur in co-infection. These observations raise questions for future research on exo-erythrocytic development of avian *Plasmodium* species, particularly in regard of the presence of cytomeres in developing exo-erythrocytic meronts of these pathogens. So far, cytomeres were not observed in tissue stages of avian malaria parasites [1, 5–7, 43].

Different strains of *P. matutinum* showed differences in virulence when inoculated to domestic canaries [5, 6, 36, 44]. High virulence and mortality were described in canaries after experimental exposure to Italian and Swiss strains [11, 38], whereas American strains seemed to be less aggressive with frequent recovery of the birds [5, 36]. Experimental inoculation with infected blood showed that domestic canaries were susceptible to a strain of pLINN1 isolated from the Thrush nightingale (*Luscinia luscinia*), but parasitaemia was low and mortality was not observed [14]. The virulence of this parasite in wild birds remains insufficiently understood. Corradetti et al. [10] reported the death of one redwing (*Turdus iliacus*) after experimental infection with an unknown lineage of *P. matutinum* and speculated that the stress of prolonged captivity keeping might have impaired the host parasite balance. This study shows that natural infection of the lineage pLINN1 is virulent and pathogenic in the free-living fieldfares without relationship to captivity stress. As the parasites matured and produced merozoites in immune cells (Fig. 1j), immune evasion from cellular immunity was suspected. The prominent presence of phanerozoite stages in several organs in comparison to the rather low parasitaemia further suggests that the examined fieldfare was not adapted to *P. matutinum* (pLINN1). The finding of large, nearly mature phanerozoites in the peripheral blood may represent an artificial contamination of the samples, as the blood films were taken from traumatically ruptured heart and lung tissue. Phagocytosis of such phanerozoites by monocytic cells, however, indicate an immune reaction *intra vitam* against phanerozoite laden endothelial cells, which are washed out from capillaries into the circulation during massive infections.

Multiple phanerozoites in samples of brain, skeletal muscle, and eye tissue, in combination with signs of vascular blockage and thrombus formation raise suspicion of an impaired vision and neuromuscular responsiveness as cause of the unexpected collision with a slow driving car. Impairment of vascular perfusion of the *Pecten oculi* due to blockage by endothelial phanerozoites may have a direct impact on the eye function itself. The *Pecten oculi* is a unique structure of the avian eye composed of multiple capillaries and larger blood vessels surrounded by pigment cells. It is assumed to serve as a nutritive organ for the avascular retina and to balance the intraocular microenvironment by regulation of pressure, pH, and physical stability of the vitreous body [45–51]. To prove this hypothesis, however, ophthalmologic examinations *intra vitam* would have been necessary.

*Plasmodium matutinum* (pLINN1) lineage is common in wild birds in Austria [4, 13] and pathogenic for local endemic birds in New Zealand [12, 52], where it was probably introduced together with their *Turdus* host species. The same lineage was recently reported to cause lethal malaria in captive African penguins *Spheniscus demersus* and Lovebirds *Agapornis roseicolli* in Italy [37, 53], and Atlantic puffins *Fratercula arctica* in Switzerland [54]. These and other exotic to Europe bird species likely are non-adapted to pLINN1 infection. It is worth to note that – similar to this case in the fieldfare—parasitaemia was low during most reported mortalities, which raises suspicion of tissue damage by exo-erythrocytic stages as cause of death. Exo-erythrocytic merogony in various organs is reported to be the most striking histologic lesion in pet and aviary birds and is particularly prominent in non-adapted hosts [55]. Furthermore, it is considered to be key pathogenic stage in experimental infections of naïve poultry flocks with *Plasmodium durae*, *Plasmodium gallinaceum*, and *Plasmodium octamerium* [56]. Mortality in most of these cases was caused by cerebral dysfunction due to the early and prominent development of exo-erythrocytic phanerozoites in endothelial cells. The brain capillaries were occluded by the swollen endothelial cells, preventing normal blood flow, and causing anoxic conditions resulting in clinical symptoms resembling cerebral stroke. Likewise, the foci of degeneration and necrosis of single fibres seen in the cardiac and skeletal muscle in close proximity to blocked and deteriorated vessels are considered a sequela of local ischaemia.

Pulmonary oedema is one of the key findings of avian malaria in captive birds and could also be confirmed for the fieldfare. Right ventricular hypertrophy (RVH) due to hypoxic pulmonary arterial hypertension is a well-documented sequela of *Plasmodium* species and *Aegyptianella pullorum* infections in poultry. It is caused by hypoxic pulmonary arterial vasoconstriction as a response to anaemia [56, 57]. The moderate left shift of the erythroid line seen in the heart blood and the bone marrow of the fieldfare indicates an increased erythropoietic activity, whose key trigger is peripheral tissue oxygen deficiency. Although the values for the PCV were not measured, these findings strongly indicate hypoxia most likely caused by anaemia and/or tissue malperfusion due to the diffuse blockage of capillary beds by phanerozoites.

Liver and spleen were only slightly enlarged, inflammatory infiltrates were mild to moderate, and parasitic stages and pigment granules were few to absent. Consequently, the organs also did not show a blackish pigmentation frequently reported in literature. This discoloration results from haemozoin accumulation in macrophages. Haemozoin is birefringent and negative on Prussian blue stain for iron, while haemosiderin is golden-brown and stains positively with Prussian blue [58]. Both pigments were present in low numbers.

Mild gastrointestinal worm infections are a common finding in necropsies of wild birds with usually little impact on the general body condition and health. Compared to the prominent lesions in brain, heart, and lung related to the *Plasmodium* infection, the low-grade helminthiasis seen in the intestine and the lung was considered of subordinate importance for the fatal roadkill. The concurrent seromucous diarrhoea, however, speaks for clinically manifest gastrointestinal disease. It may have been caused by the intestinal nematodiasis with or without an undetected gastrointestinal microbial co-infection and was possibly facilitated by the malarial infection [59].

Clarification of a possible concurrent microbial disease with special emphasis on West Nile Virus, Usutu Virus, and *Chlamydiaceae* was of particular interest, as the inflammatory patterns and organ distribution of lesions seen corresponded to patterns of these diseases described in literature [55].

Testing for microbial co-infections was negative in the extraintestinal compartment, and NGS was negative for both the extraintestinal and the intestinal compartment. This shows that the moderate to prominent inflammatory infiltrates observed in several organs outside the gastrointestinal tract were related to the malaria infection.

Like many parasites of the phylum Apicomplexa, infections with *Plasmodium* species trigger a predominantly mononuclear inflammatory reaction consisting of various portions of lymphocytes, plasma cells, and macrophages/histiocytes. Depending on the host immunity and the pathogenicity of the lineages the inflammatory lesions vary from mild to dramatic. Severe reactions may be mistaken for lymphoid neoplasia [55]. The findings in the SSS and PWP of the spleen suggest a prominent reaction of the highly phagocytic reticular ellipsoid associated cells and an increased reactivity of the B-cells in the surrounding peri ellipsoid lymphocytic sheath. Interpretation of the eosinophilia seen in the heart blood is limited as the nature of this cell type differs from mammals and remains not fully understood in avian species [60–63]. Prominent exo-erythrocytic development, phagocytosis of phanerozoites seen in circulating monocytes and concurrent successful maturation of phanerozoites within leukocytes raises suspicion of both an active antiparasitic immune response and immunoevasive mechanisms playing a role in the pathogenesis of this malaria case. As this report is based on a single natural infection of unknown history with a co-infection with helminths, these considerations remain speculative. Targeted immunological studies under experimental conditions would be of interest to clarify a true correlation of these findings to an infection with *P. matutinum*, to characterize the anti-parasite response profile of the host immune system as reported for other apicomplexan pathogens [64], and to elucidate possible immunoevasive strategies which allowed the parasite to escape from cellular immunity.

This study shows that *P. matutinum* (pLINN1) is an aggressive malaria parasite, which can develop even in immune cells and is dangerous for non-adapted wild birds. This study supports formerly fragmental observations that avian *Haemamoeba* malaria parasites can develop and produce merozoites in monocytes and macrophages [5, 6]. The true role of this infection as a potential threat for wildlife bird populations remains to be investigated.

Millions of birds are killed on roads due to collisions with vehicles each year [65]. This study shows that severe malaria infections likely contribute to such mortalities. Interestingly, examination of roadkill juvenile chaffinches *Fringilla coelebs* revealed exceptionally high (up to 7%) parasitaemia of *Haemoproteus* species in comparison to the same age bird species, which were mist-netted at the same area [6]. This indicates possible involvement of avian haemoproteosis in road mortalities. This is not unexpected due to recent findings of megalomeronts of *Haemoproteus* parasites in brain of naturally infected birds [3]. This limited available information suggests that avian haemosporidian infections are worth more attention as agents of avian diseases. The described case of severe *P. matutinum* (pLINN1) malaria in the fieldfare emphasizes the importance of further studies on exo-erythrocytic stages of haemosporidian parasites as potential underestimated cause of fatal disease in wild bird populations in general, and in roadkill in particular.

## Conclusions

This study reports an active natural *P. matutinum* (lineage pLINN1) infection in the fieldfare. The exo-erythrocytic development of the pLINN1 infection was described for the first time. It consisted of phanerozoites, which matured in circulating immune cells (monocytes/macrophages and granulocytes) as well as in capillary endothelial cells of multiple organs. Conspicuous vacuolization of the cytoplasm in phanerozoites and erythrocytic meronts is a characteristic feature of the merogony during *P. matutinum* infection. The findings of multiple phanerozoites in all samples of the brain as well as skeletal muscle and eye tissue in combination with signs of vascular blockage and thrombus formation suggested an impaired vision and neuromuscular responsiveness as cause of the unexpected and fatal collision with a slow-moving car. Wild birds suffer from malaria in Europe, but the true level of populational damage still needs to be identified in wildlife. This case emphasizes the importance of further studies on exo-erythrocytic stages of haemosporidian parasites as potential underestimated cause of fatal disease in wild bird populations.

## Data Availability

Voucher preparations of blood and tissue stages of *P. matutinum* (pLINN1) were deposited at Nature Research Centre, Vilnius. The data generated during this study are included in this published article.
